# Author Correction: Pharmacological markers of HIV prevention for oral pre-exposure prophylaxis in men who have sex with men

**DOI:** 10.1038/s41467-026-73598-9

**Published:** 2026-05-20

**Authors:** Sara Iannuzzi, Malin Müller, Yifan Yu, Lanxin Zhang, Craig W. Hendrix, Robert R. Bies, Max von Kleist

**Affiliations:** 1 Projektgruppen, Robert-Koch Institute Berlin, Berlin, Germany; 2 Department of Mathematics and Computer Science, Freie Universität Berlin, Berlin, Germany; 3 Division of Pharmacokinetics, Pharmacodynamics and Systems Pharmacology, School of Pharmacy and Pharmaceutical Sciences, University at Buffalo, Buffalo, NY USA; 4 Department of Medicine (Clinical Pharmacology) and Pharmacology and Molecular Sciences, Johns Hopkins University School of Medicine, Baltimore, MD USA

**Keywords:** HIV infections, Computational models, Stochastic modelling, Pharmacodynamics, Pharmacokinetics

Correction to: *Nature Communications* 10.1038/s41467-026-72907-6, published online 10 May 2026

In the version of this article initially published, there was a typographical error in the Fig. 4b *x*-axis label, where in the text now reading “Δt = time of exposure – time of first dose (hours),” “hours” appeared originally as “days.” The figure is now updated in the HTML and PDF versions of the article.

