# Supplementary material for: Pharmacological markers of HIV prevention for oral pre-exposure prophylaxis in men who have sex with men
Source: Nat Commun. 2026 May 10;17:4213. doi: 10.1038/s41467-026-72907-6 (PMC13157487; doi:10.1038/s41467-026-72907-6)
Supplement: Supplementary file 1 — Supplementary Information [file 41467_2026_72907_MOESM1_ESM.pdf]

# Supplementary Materials

## Comparison of TDF and FTC pharmacokinetic parameters stratified by sex

| Model | Male + Female | | Male | | Female | |
|---|---|---|---|---|---|---|
| Parameter | Final Estimate (RSE%) | BSV %CV (RSE%) | Final Estimate (RSE%) | BSV %CV (RSE%) | Final Estimate (RSE%) | BSV %CV (RSE%) |
| CL (L/h) | 51.6 (5%) | 15.5 (29%) | 56.1 (7%) | 15.1 (31%) | 53 (8%) | 21% (65%) |
| V2(L) | 706 (15%) | 19.3% (75%) | 764 (17%) | | 647 (30%) | 20.5% (162%) |
| KA (/h) | 0.518 (38%) | 90.6% (25%) | 0.668 (58%) | 123.3% (37%) | 0.654 (48%) | 117.9% (40%) |
| Q (L/h) | 11 (25%) | 86.2% (24%) | 13.7 (41%) | 87.6% (29%) | 16.5 (79%) | 98.2% (83%) |
| V3 (L) | 478 (13%) | 32.6% (25%) | 424 (16%) | 25.8% (120%) | 516 (44%) | 85.6% (149%) |
| $k_{\text{in}-\text{TFV}-PBMC}$ (/h) | 0.0075 (8%) | 29.4% (19%) | 0.0073 (16%) | 29.1% (39%) | 0.0086 (21%) | 33.3% (23%) |
| $k_{\text{out}-\text{PBMC}}$ (/h) | 0.0098 (5%) | 8% (74%) | 0.0097 (10%) | 9.1% (121%) | 0.0102 (5%) | |
| $TVCL$ | 0.323 (35%) | | 38.3% (6%) | | 42.9% (5%) | |
| PROP (Plasma TFV) | 43.2% (3%) | | 26.9% (6%) | | 33.4% (4%) | |
| PROP (PBMC TFV-dp) | 30.5% (3%) | | | | | |

**Table 1: Comparison of pharmacokinetic parameters for the TFV PK model across three models derived from datasets stratified by sex.** RSE: relative standard errors, BSV: between-subject variability, CV: coefficient of variation. Source data are provided as a Source Data file.

| Individual | Mutation | Plasma TVF | TVF-DP DBS | Interpretation |
|---|---|---|---|---|
| E1 | | LLOQ < 10 ng/mL | BLQ | Not taking the drug |
| E2 | | BLQ | BLQ | Not taking the drug |
| **E3** | **M184I** | **≥ 10 - < 40 ng/mL** | **> LLOQ - 350 fmol/punch** | **Taking some drug (possible white coating)** |
| E4 | | BLQ | > LLOQ - 350 fmol/punch | Stopped taking the drug |
| E5 | | BLQ | BLQ | Not taking the drug |
| E6 | | BLQ | BLQ | Not taking the drug |
| E7 | | BLQ | BLQ | Not taking the drug |
| **E8** | | **> 40 ng/mL** | **> LLOQ - 350 fmol/punch** | **Taking some drug (possible white coating)** |
| E9 | | BLQ | BLQ | Not taking the drug |
| E10 | | BLQ | BLQ | Not taking the drug |
| E11 | | BLQ | BLQ | Not taking the drug |
| E12 | | BLQ | BLQ | Not taking the drug |
| E13 | M184V | BLQ | > LLOQ - 350 fmol/punch | Stopped taking the drug |
| E14 | | BLQ | BLQ | Not taking the drug |
| E15 | | BLQ | > LLOQ - 350 fmol/punch | Stopped taking the drug |
| **E16** | **K65R** | **> 40 ng/mL** | **≥ 1250 fmol/punch** | **Taking some drug** |
| E17 | | BLQ | BLQ | Not taking the drug |
| E18 | | BLQ | BLQ | Not taking the drug |
| E19 | | BLQ | > LLOQ - 350 fmol/punch | Stopped taking the drug |
| E20 | | BLQ | BLQ | Not taking the drug |
| E21 | | BLQ | BLQ | Not taking the drug |
| **E22** | | **> 40 ng/mL** | **> LLOQ - 350 fmol/punch** | **Taking some drug (possible white coating)** |
| E23 | | BLQ | BLQ | Not taking the drug |
| E24 | | BLQ | BLQ | Not taking the drug |
| E25 | M184V | BLQ | BLQ | Not taking the drug |
| E26 | | BLQ | BLQ | Not taking the drug |
| E27 | | BLQ | > LLOQ - 350 fmol/punch | Stopped taking the drug |
| E28 | | BLQ | > LLOQ - 350 fmol/punch | Stopped taking the drug |
| E29 | | BLQ | BLQ | Not taking the drug |
| E30 | | BLQ | BLQ | Not taking the drug |
| E31 | | BLQ | BLQ | Not taking the drug |
| E32 | | BLQ | BLQ | Not taking the drug |
| E33 | | BLQ | BLQ | Not taking the drug |
| **E34** | | **> 40ng/mL** | **≥700 - < 1250 fmol/punch** | **Taking some drug** |
| E35 | | BLQ | BLQ | Not taking the drug |
| E36 | | BLQ | > LLOQ - 350 fmol/punch | Stopped taking the drug |
| E37 | | BLQ | > LLOQ - 350 fmol/punch | Stopped taking the drug |
| **E38** | | **> 40 ng/mL** | **> LLOQ - 350 fmol/punch** | **Taking some drug (possible white coating)** |
| E39 | | BLQ | > LLOQ - 350 fmol/punch | Stopped taking the drug |

**Table 2: TFV and TFV-DP levels in infected individuals in the HPTN 083 study**. Adopted from the HPTN 083 trial[1] (supplementary appendix therein). Individuals E3, E16, E34 (in bold) were classified as drug-detected after further analysis[2] (supplementary appendix therein to identify their TFV-DP levels as clearly above 200 fmol/punch (approximately 1 dose/week)[3–5]. Additonally E8, E22 and E38 (in bold) had both TFV and TFV-DP levels above LLOQ, indicating some long- and short-term adherence. Thus, out of the 39 infected individuals in HPTN 083, 6 were classified as part of the drug-detected group (taking some drug). This may include some individuals with white coat dosing (very low TFV-DP concentrations in DBS, but high plasma TFV), consistent with recent PrEP re-initiation just before sample collection. Among the non-adherent individuals some were classified as stopped taking drug (detectable TFV-DP in DBS, but no plasma TFV), suggesting cessation of dosing several days prior to sampling, in which case TFV-DP in DBS remains detectable due to its long half-life. There are some individuals who have PrEP resistant mutations (FTC and TFV-DP resistance mutations M184I/V and K65R respectively[6]), which may be due to transmitted resistance or de novo selection. Source data are provided as a Source Data file.

| Study | Intervention arm | | | | | HIV-1 seroconverters | | | |
|---|---|---|---|---|---|---|---|---|---|
| | Matrix/Analyte | Participants selection | No Drug Cut-off | No Drug Interpretation | Source | Matrix/Analyte | No Drug Cut-off | No Drug Intepretation | Source |
| IPERGAY | Plasma TFV | Sequential (57%) | ≤ LLOQ (0.1 ng/mL) | < 1/wk | Fig S2A (month 1-8) in [13] | Plasma TFV | ≤ LLOQ (0.1 ng/mL) | < 1/wk | Fig 2A in [13] |
| iPrEX | Plasma TFV | 3:1 matched controls (15%) | ≤ LLOQ (10 ng/mL) | ≤ ~4/wk | Fig S7 in [19] | FTC-TP in PBMCs | ≤ LLOQ (10 ng/mL) | ≤ ~4/wk | Fig 4A and Fig S7 in[19] |
| HPTN 083 | Plasma TFV | Random (17%) | ≤ LLOQ (0.31 ng/mL) | < 1/wk | Main text in [25] | DBS TFV-DP, Plasma TFV | Plasma TFV < 40 ng/mL or DBS TFV-DP < 200 fmol/punch (See Table S2) | < 1/wk (See Table S2) | Supp. File 6 in [25] |
| PURPOSE 2 | DBS TFV-DP (week 8-52) | Random (10%) | ≤ 350 fmol/punch | < 2/wk¶ | Figure 3B in [27] | DBS TFV-DP | ≤ 350 fmol/punch | < 2/wk¶ | In text of [27] |
| DISCOVER | DBS TFV-DP | Random (10%) | ≤ 350 fmol/punch | < 2/wk¶ | Supplementary Figure S14 in [26] | DBS TFV-DP | ≤ 350 fmol/punch | < 2/wk¶ | Supplementary Figure S2 in [26] |

**Table 3: Overview of studies and available adherence markers.** Adherence data refer to the indicated source datasets and were used to dichotomize clinical data. TFV: tenofovir; TFV-DP: tenofovir diphosphate; DBS: dried blood spots; FTC-TP: emtricitabine triphosphate; PBMC: peripheral blood mononuclear cells. ¶ Cannot differentiate 1 dose/wk from BLQ. Source data are provided as a Source Data file.

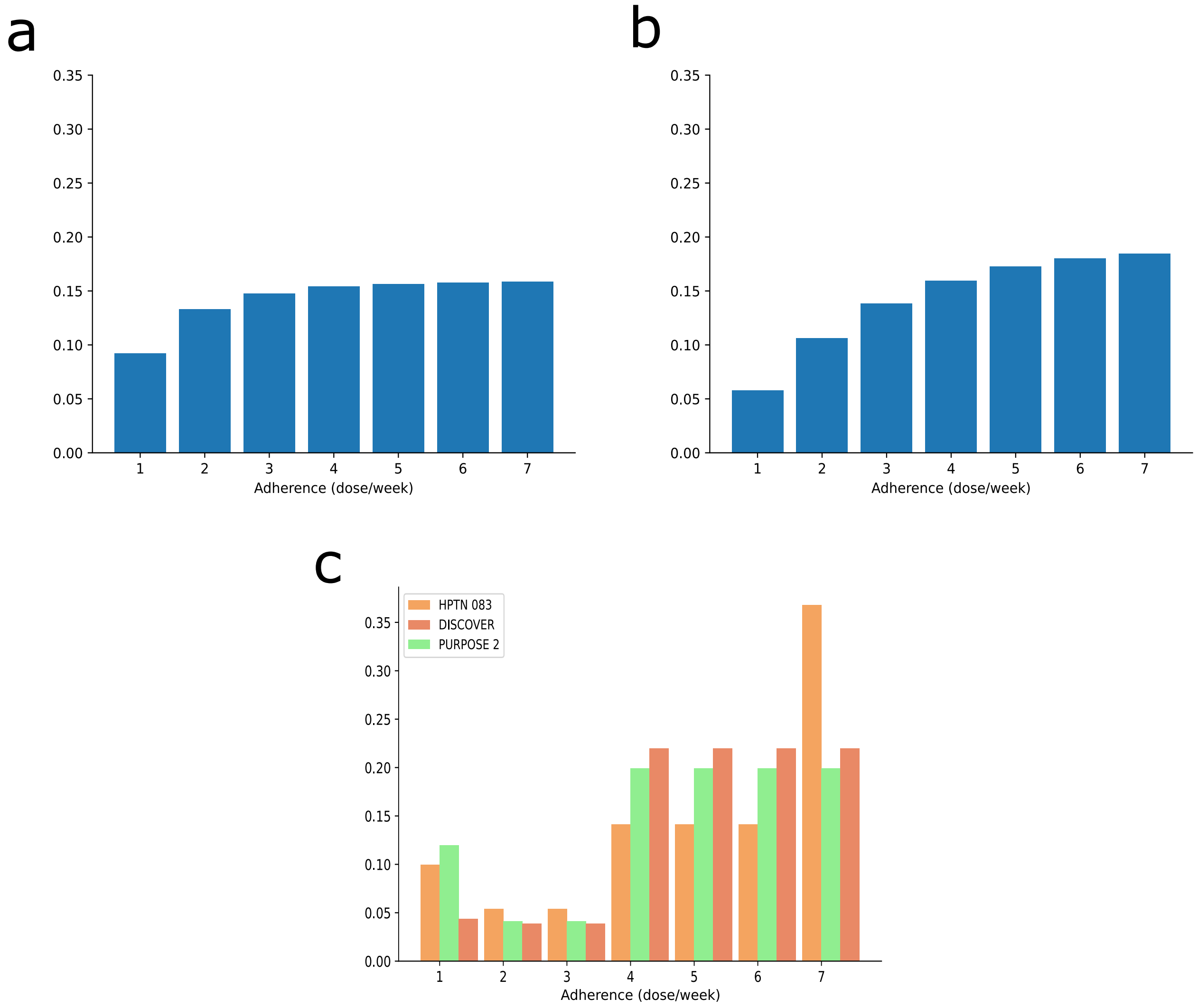


**Figure 1: Adherence Levels Across Trials.** (**a**) For each clinical study, simulated dosing frequencies for trial simulation (see Methods) were set to match the adherence levels in the original study. Computed probability of plasma TFV detection at LLOQ: 0·001 $\mu$M (corresponding to the detection limit in IPERGAY) using the population pharmacokinetic model. (**b**) Computed probability of plasma TFV detection LLOQ 0·035 $\mu$M (the detection limit in iPrEX). (**c**) Reported probability adherence levels for HPTN 083, DISCOVER and PURPOSE 2 clinical trials. If an adherence strata involved multiple dosing frequencies (i.e. $> 4$ doses, we assigned equal probability to each dosing frequence, i.e. 5, 6, 7 doses/week). For HPTN 083, BLQ random samples were excluded from our analysis and probabilities re-normalized (Figure S1 in[1]). LLOQ: Lower limit of quantification. Source data are provided as a Source Data file.

## Sensitivity of findings to choice of pharmacokinetic matrices.

It has been reasoned that systemic CD4$^+$ T cells (which are reflected by the PBMC compartment) and tissue resident CD4$^+$ T cells may encompass different drug concentrations due to limiting drug distribution across tissues[7]. It has therefore been speculated for heterosexual exposure of cis-women that both systemic, as well as local tissue drug concentrations contribute to F/TDF PrEP efficacy[8]. Therefore, a (model-)testable question may be whether a mix of pharmacokinetic matrices (rectal tissue and PBMC) may be used to predict PrEP efficacy against RAI in MSM?

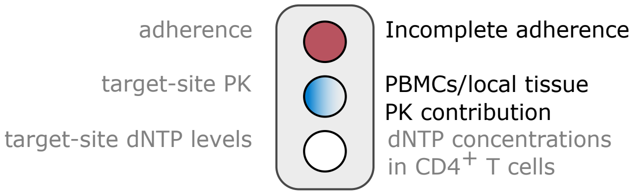


**Figure 2: Hypothesis testing scheme for pharmacokinetic matrices.** Different relative contributions of colorectal tissue and PBMC pharmacokinetics were simulated (blue light). Adherence (red light) was incomplete according to trial-specific adherence information (Suppl. Fig. 1). In all simulations (white light), we used dNTP concentrations in CD4$^+$ T-cells to predict drug potency.

### Could both systemic and local tissue drug concentrations contribute to PrEP efficacy for RAI?

We designed a computational experiment, where we modelled PrEP efficacy as a function of both markers using fixed mixing weights to generate a composite PK profile representing target-site concentrations. In our computational experiments 50, 30 and 10 % of the composite PK-profile where determined by colo-rectal tissue PK, with the remaining proportion being PBMC pharmacokinetics, as illustrated by the light switch in Suppl. Fig. 2. Akin to the main manuscript, we then simulated the different clinical trials and computed empirical $P$-values by comparing the distribution of the number of infections obtained from clinical data vs. our simulations assuming the the above-mentioned mixed pharmacokinetic marker, as illustrated by the light-switch annotation in Suppl. Fig. 4.
For HPTN 083, the infection distributions predicted from the mechanistic simulations remained statistically distinct from those derived from clinical data, when local tissue pharmacokinetics contributed 50% or more to the composite target-site pharmacokinetic marker. For the DISCOVER trial, if local tissue pharmacokinetics contributed 10% or more to the putative target-site pharmacokinetics, mechanistic simulations yielded trial outcomes that were statistically distinct from clinical-data derived outcomes, Suppl. Table 4.
In summary, based on both informative trials (HPTN 083 and DISCOVER), it remains unlikely that drug concentrations in colo-rectal tissue can be used, even if only partially, to predict PrEP efficacy against RAI in MSM.

| Scenario | Data-driven | 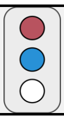 | 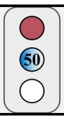  | 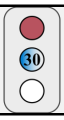  | 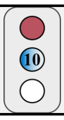  | 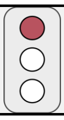 | Study |
|---|---|---|---|---|---|---|---|
| PrEP efficacy<br>$Inf_{sim}$<br>P-value | 88.4 (40, 100)<br>1 (0, 4)<br>- | 70.8 (19, 98)<br>3 (0, 9)<br>$3.3 \times 10^{-1}$ | 81.5 (33, 100)<br>1 (0, 4)<br>$6.3 \times 10^{-1}$ | 82.4 (36, 100)<br>1 (0, 4)<br>$6.8 \times 10^{-1}$ | 83.4 (39, 100)<br>1 (0, 4)<br>$7.1 \times 10^{-1}$ | 83.5 (38, 100)<br>1 (0, 4)<br>$7.2 \times 10^{-1}$ | **IPERGAY** |
| PrEP efficacy<br>$Inf_{sim}$<br>P-value | 96.5 (93, 99)<br>5 (1, 10)<br>- | 77.1 (65, 87)<br>33 (19, 50)<br>$1. \times 10^{-5}$ | 90.8 (83, 96)<br>13 (6, 21)<br>$4.7 \times 10^{-2}$ | 92.2 (85, 97)<br>11 (4, 18)<br>$9.8 \times 10^{-2}$ | 93.1 (86, 98)<br>9 (3, 17)<br>$1.5 \times 10^{-1}$ | 93.3 (87, 98)<br>9 (3, 16)<br>$1.8 \times 10^{-1}$ | **HPTN 083** |
| PrEP efficacy<br>$Inf_{sim}$<br>P-value | 87.1 (71, 97)<br>3 (0, 6)<br>- | 73.9 (42, 95)<br>6 (1, 11)<br>$2.2 \times 10^{-1}$ | 87.5 (64, 100)<br>2 (0, 5)<br>$6.9 \times 10^{-1}$ | 89.1 (67, 100)<br>2 (0, 5)<br>$7.5 \times 10^{-1}$ | 90.0 (69, 100)<br>1 (0, 4)<br>$7.9 \times 10^{-1}$ | 90.5 (71, 100)<br>1 (0, 4)<br>$8.1 \times 10^{-1}$ | **iPrEx** |
| PrEP efficacy<br>$Inf_{sim}$<br>P-value | 84.2 (51, 97)<br>3 (0, 7)<br>- | 80.5 (51, 98)<br>4 (0, 10)<br>$3.5 \times 10^{-1}$ | 90.0 (68, 100)<br>2 (0, 5)<br>$7.2 \times 10^{-1}$ | 91.0 (70, 100)<br>1 (0, 5)<br>$7.7 \times 10^{-1}$ | 91.6 (72, 100)<br>1 (0, 4)<br>$8. \times 10^{-1}$ | 91.8 (71, 100)<br>1 (0, 4)<br>$8.1 \times 10^{-1}$ | **PURPOSE 2** |
| PrEP efficacy<br>$Inf_{sim}$<br>P-value | 98.7 (97, 99)<br>2 (0, 6)<br>- | 74.6 (62, 85)<br>39 (18, 68)<br>$1. \times 10^{-5}$ | 91.2 (83, 97)<br>13 (4, 24)<br>$1.1 \times 10^{-2}$ | 92.9 (86, 98)<br>10 (3, 20)<br>$3.2 \times 10^{-2}$ | 93.9 (87, 98)<br>8 (2, 17)<br>$5.6 \times 10^{-2}$ | 94.3 (88, 99)<br>8 (2, 16)<br>$7.1 \times 10^{-2}$ | **DISCOVER** |

**Table 4: Statistical assessment of mixed pharmacokinetic marker in regards to clinical data**. Each column represents simulation outcomes derived from mechanistic modeling and simulation of the respective clinical trials under the indicated hypotheses (light-switch annotation). The first column: (Data-driven) denotes outcomes obtained from the clinical data. The rows present the outcomes for the different clinical trials: Average PrEP efficacy (95% CI), the average number of infections (95% CI) when the distinct trials were simulated (see Clinical Trial Simulations in Methods). For each hypothesis, a one-sided $P$-value was computed. The $P$-values test for differences in clinical trial outcomes (number of infected individuals) between the data-driven and hypothesis driven approaches under the null hypothesis ($\mathcal{H}_0$: identical distributions) versus the alternative ($\mathcal{H}_1$: different distributions), computed by running $10^5$ simulations per pair. The light-switch annotation summarizes mechanistic scenarios from left to right: (i) tissue concentrations account for the pharmacokinetic effect (0% PBMC), (ii) the pharmacokinetic effect marker is based on a 50% tissue and 50% PBMC contribution (i.e. the arithmetic average), (iii) is based on a 30% tissue and 70% PBMC contribution, (iv) a 10% tissue, 90% PBMC contribution, and (v) is based on concentrations in PBMC only. In all scenarios, drug potency was estimated based on intracellular dNTP levels in CD4+ T cells and adherence was simulated according to the respective adherence strata from the clinical trials, see Suppl. Fig. 1. Source data are provided as a Source Data file. PBMC: peripheral blood mononuclear cells, dNTP: deoxynucleoside triphosphate.

## Sensitivity of findings with regards to virus exposure routes.

While results in the main manuscript were based on simulations assuming that all relevant exposures occurred via receptive anal intercourse (RAI), we assessed the robustness of our findings when other routes of exposure, as well as differential PrEP efficacy (and thereby relevant PK markers) for these

exposure routes were considered. Three primary exposure routes are associated with sexual HIV transmission among men who have sex with men (MSM): receptive anal intercourse (RAI), insertive anal intercourse (IAI), and oral intercourse. Below, we extended our modeling framework to account for viral exposure via all relevant routes.

## Modeling different viral exposure routes in MSM

We extended our modeling framework to account for viral exposure via different routes. This required to (i) estimate the exposure-specific inoculum and (ii) the relative frequency of different exposure routes. These two factors determine the overall importance of an exposure route to HIV transmission in MSM. Lastly, the efficacy of PrEP in inhibiting transmission via different exposure routes is directly related to pharmacokinetic matrices that predict PrEP efficacy. We will test whether tissue concentrations may predict PrEP efficacy in MSM under extreme assumptions that would favor this PK matrix.

**Exposure-specific inoculum.** We extended a previously developed framework[9] to account for IAI. In our framework, the number of transmitted viruses that successfully translocate to a physiological site that is permissive to infection is modeled as a random variable drawn from a binomial distribution $\mathcal{B}(VL, r_{\text{exposure}})$, where $VL$ denotes the viral load in the donor (log-normal distributed with values derived from sero-converter cohorts) and $r_{\text{exposure}}$ is the per-virion translocation probability, or success probability, associated to a specific exposure route (see derivation and parameterization in[9], Supplementary Text S1 therein). The success probabilities $r_{\text{RAI}}$ and $r_{\text{IAI}}$ are then estimated to reproduce empirically observed infection probabilities for unprotected sexual intercourse[10,11], as summarized in Supplementary Table 5 and depicted in Supplementary Figure 3). The resulting parameter estimates were $3.7 \times 10^{-3}$ and $1.3 \times 10^{-4}$ for $r_{\text{RAI}}$ and $r_{\text{IAI}}$ respectively.

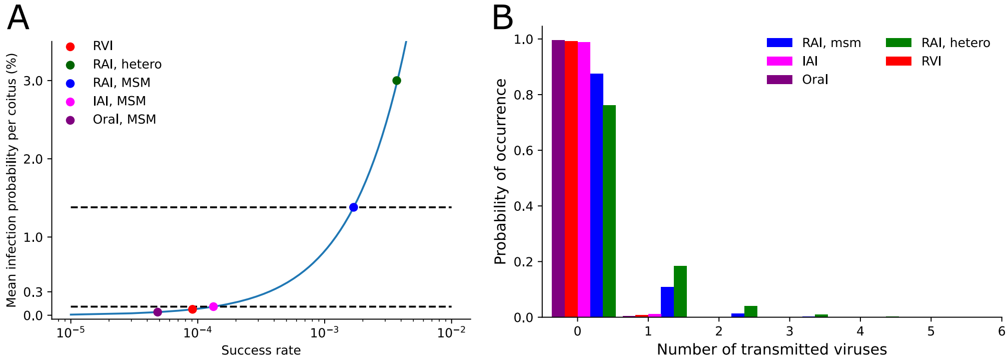


**Figure 3: Infection probabilities and virus translocation probabilities for different virus exposure routes.** (**a**) Relation between success rate (probability of virus to translocate to a physiological site where it can initiate infection) and the mean infection probability per coitus, for different exposure routes[10,11]. (**b**) Inoculum size distributions per exposure route. RVI: Receptive Vaginal Intercourse; RAI: Receptive Anal Intercourse; IAI: Insertive Anal Intercourse; Oral: oral sexual intercourse. Source data are provided as a Source Data file.

| Exposure type | $\hat{P}$(inf) per coitus (%) | Contribution to infection (%) |
|---|---|---|
| RAI | 1.38 (1.02–1.86) | 69 (59–79) |
| IAI | 0.11 (0.04–0.28) | 28 (19–38) |
| Oral intercourse | ≤0.04 (0–0.04) | 2 |

**Table 5: Exposure-route specific HIV transmission probabilities and relative contribution to infection among MSM.** Per-act average HIV transmission probabilities ($\hat{\mathbf{P}}(\mathbf{inf})$), and relative contribution of exposure route to HIV transmission, e.g. $P(\text{RAI} \mid \text{Inf}, \varnothing)$ (= the conditional probability that an infection is attributed to RAI exposure, in the absence of pharmaceutical intervention $\varnothing$), as stated in[10,11]. Reported values represent the median and 95% confidence intervals. RAI: receptive anal intercourse; IAI: insertive anal intercourse. Source data are provided as a Source Data file.

In Supplementary Figure 3B, the resulting inoculum size distribution is depicted. As expected, RAI implies larger inoculum sizes, reflecting both high viral loads in donor fluid (semen) and a weak physiological barrier consisting of only one epithelial layer separating the transmission fluid (semen) from entering the exposed individual, and eventually HIV target cells.

**Relative frequency of exposure route.** Next, we estimated the relative frequency of RAI and IAI exposures ($\pi_{\text{RAI}}$ and $\pi_{\text{IAI}}$) in MSM, analogously to previous work on HIV transmission in heterosexual women[12]. We did not consider oral exposures in our simulations, since the reported contribution to HIV transmission is small in comparison to the other exposure routes, see Suppl. Table 5. In order to gauge the frequency of RAI and IAI exposures for subsequent model tests, we solved the following equation for $\pi_{\text{RAI}}$ and $\pi_{\text{IAI}}$ using data depicted in Supplementary Table 5:

$$P(\text{RAI} \mid \text{Inf}, \varnothing) = \frac{\pi_{\text{RAI}}\, \hat{P}(\text{Inf} \mid \text{RAI}, \varnothing)}{\pi_{\text{RAI}}\, \hat{P}(\text{Inf} \mid \text{RAI}, \varnothing) \;+\; \pi_{\text{IAI}}\, \hat{P}(\text{Inf} \mid \text{IAI}, \varnothing)}. \tag{1}$$

where $P(\text{RAI} \mid \text{Inf}, \varnothing)$ denotes the relative contribution of RAI to HIV infection, $\hat{P}(\text{Inf}|\text{IAI}, \varnothing)$, $\hat{P}(\text{Inf}|\text{RAI}, \varnothing)$ denote the average per-act infection probabilities for unprotected insertive- vs. receptive anal intercourse, respectively, with data depicted in Suppl. Table 5.

Results in the main manuscript relate to simulation considering only RAI. Therefore, to put our model and results in the main manuscript to the test, we made assumptions that would maximize the impact of IAI: We used the lower bound of the 95% confidence interval on $\hat{P}(\text{Inf})$ for RAI and the upper bound for IAI, thus maximizing the relative contribution of IAI. Given the negligible contribution of oral exposure, the median contributions of RAI and IAI were normalized. This yielded values of $\pi_{\text{RAI}} = 0.4$ and $\pi_{\text{IAI}} = 0.6$. Using the equation above it is then possible to compute the fraction of infections that would be attributed to the exposure route, deriving at $\text{P}(\text{IAI} \mid \text{Inf}, \varnothing) = 0.3$ and $\text{P}(\text{RAI} \mid \text{Inf}, \varnothing) = 0.7$. These values can be used to compute average clinical trial PrEP efficacies against mixed exposures, as derived in Suppl. eqs. (2)-(9).

### Impact of virus exposure on PrEP adherence-efficacy relationship

Foremost, we used our model to assesse whether the HIV exposure route has an impact on the adherence-efficacy relationship (akin to Fig. 2c-d, main manuscript). We computed the adherence-efficacy relationship for RAI and IAI in isolation (Suppl. Fig. 4A-B, D-E), in cases where drug concentrations in PBMC (panels A-B) vs. in tissues (panels D-E) predict PrEP efficacy. Our simulations clearly indicate that the adherence-efficacy relationship is not exposure-route specific, but rather depends on the pharmacokinetic matrix used (tissue vs. PBMC). Consequently, if the same pharmacokinetic matrix was used for RAI and IAI, simulations where 60% of exposures where IAI and 40% RAI ($\pi_{\mathrm{IAI}} = 0.6$, $\pi_{\mathrm{RAI}} = 0.4$; panels C and F) yielded the same adherence-efficacy relationships as 100% RAI or 100% IAI.
Since insufficient penile TFV-DP and FTC-TP concentration measurements (possibly important for modelling IAI) are available for modeling, we will subsequently test the robustness of our main findings (tissue concentrations do not estimate prophylactic efficacy for F/TDF PrEP in MSM) with regards to various (best-case, worst-case) assumptions regarding the prophylactic efficacy of F/TDF in IAI.

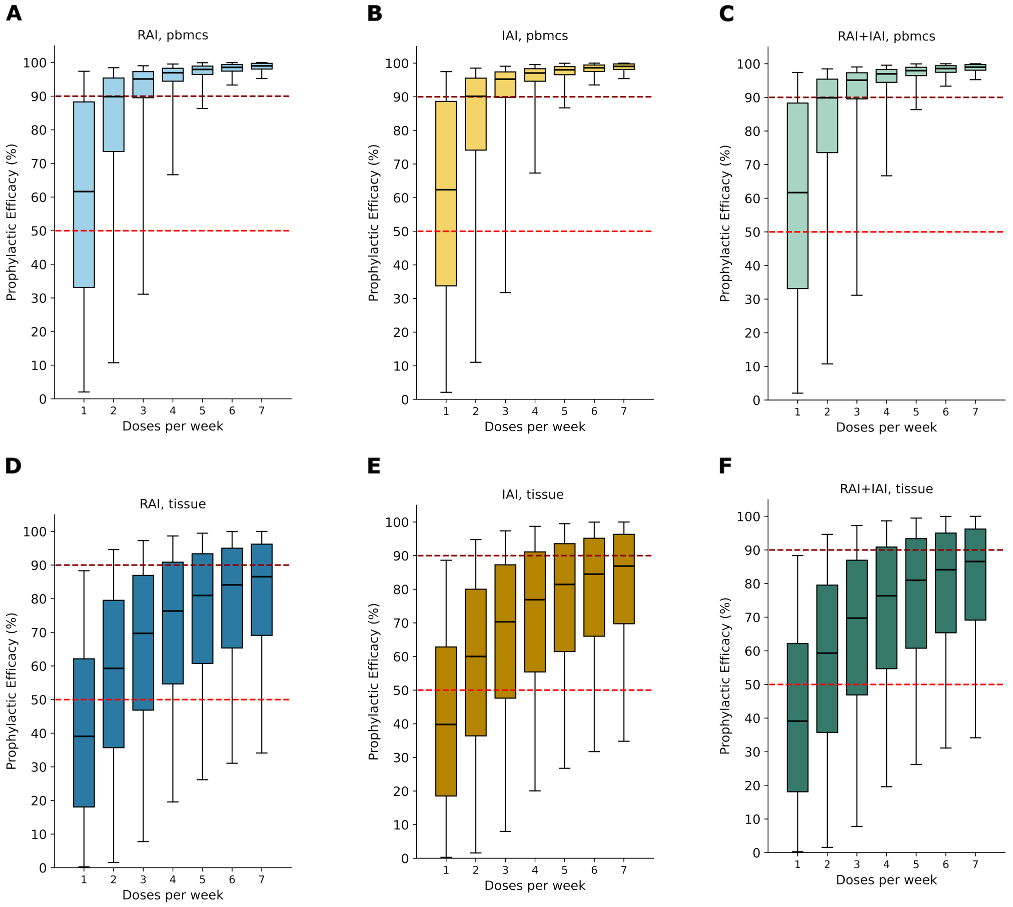


**Figure 4: Adherence-efficacy relationship for different exposure routes.** Prophylactic efficacy simulations for 1000 virtual patients stratified by average weekly pill intake, based on 348,000 time points per regimen and considering RAI exposures (panel **a, d**), IAI exposures (panel **b, e**) and mixed exposures (40% RAI and 60% IAI, panel **c, f**). The top panels (**a-c**) show simulation results when TFV-DP and FTC-TP pharmacokinetics in PBMC were used to predict drug effect, whereas the lower panels (**d-f**) show results when TFV-DP and FTC-TP pharmacokinetics in tissues where used to predict drug effect. Central lines in the boxplots represent the median PrEP efficacy, boxes depict the inter-quartile ranges, and whiskers encompass the 95% percentile ranges. Source data are provided as a Source Data file.

## Does a mix of exposure routes allow predicting PrEP efficacy from tissue pharmacokinetics?

Finally, to evaluate the potential impact of insertive anal intercourse (IAI) on overall efficacy estimates, we implemented a framework in which route-specific efficacies (RAI, and IAI) were weighted by the posterior probabilities of infection attributable to each exposure type, i.e. $P(\text{IAI} \mid \text{Inf}, \varnothing) = 0.3$ and $P(\text{RAI} \mid \text{Inf}, \varnothing) = 0.7$ (see next section). Since insufficient penile TFV-DP/FTC-TP con-

centrations are available for modeling relevant local tissue concentrations during IAI and local tissue concentrations under-predict clinical trial efficacy against RAI (main manuscript, Fig. ??), we devised an extreme-case scenario where F/TDF PrEP efficacy for IAI was fixed at its theoretical maximum ($\varphi_{\text{IAI}} = 100\%$). This computational experiment would allow to test whether the hypothesis where local tissue concentrations predict RAI can be rescued under extreme-case assumptions.

In Suppl. Fig. 5 we depict clinical data-derived PrEP efficacies (Data-driven; pink violin plots) vs. simulated PrEP efficacies for the distinct trials assuming 100% RAI exposure using either PBMC (light orange violin plots) or colorectal tissue PK (light blue violin plots), according to the results presented in the main manuscript. In addition, we depict the maximum lifting of overall PrEP efficacy for mixed exposures, i.e. when tissue or PBMC PK was used for modelling RAI efficacy, and considering a 30% contribution of the IAI route to HIV transmission (compare Suppl. Table 5), which is entirely prevented by F/TDF ($\varphi_{\text{IAI}} = 100\%$, darker orange and dark blue violin plots). Notably, simulations in which PrEP efficacy against RAI was predicted from tissue concentrations and when drug efficacy against IAI was 100%, clinical trial simulations still yielded significantly distinct outcomes to the actual clinical data (HPTN 083 and DISCOVER trial, numbers above the violin plots), in line with the main text.

Overall, these computational experiments suggest that (i) the contribution of IAI to clinical trial efficacy is, at most, moderate. Moreover, (ii) the simulations suggest that PBMC pharmacokinetics provide a more suitable marker for characterizing the adherence–efficacy relationship than local tissue concentrations, even when further splitting the data by exposure route and making the most extreme assumptions $\varphi_{\text{IAI}} = 100\%$ in favor for the local tissue biomarker.

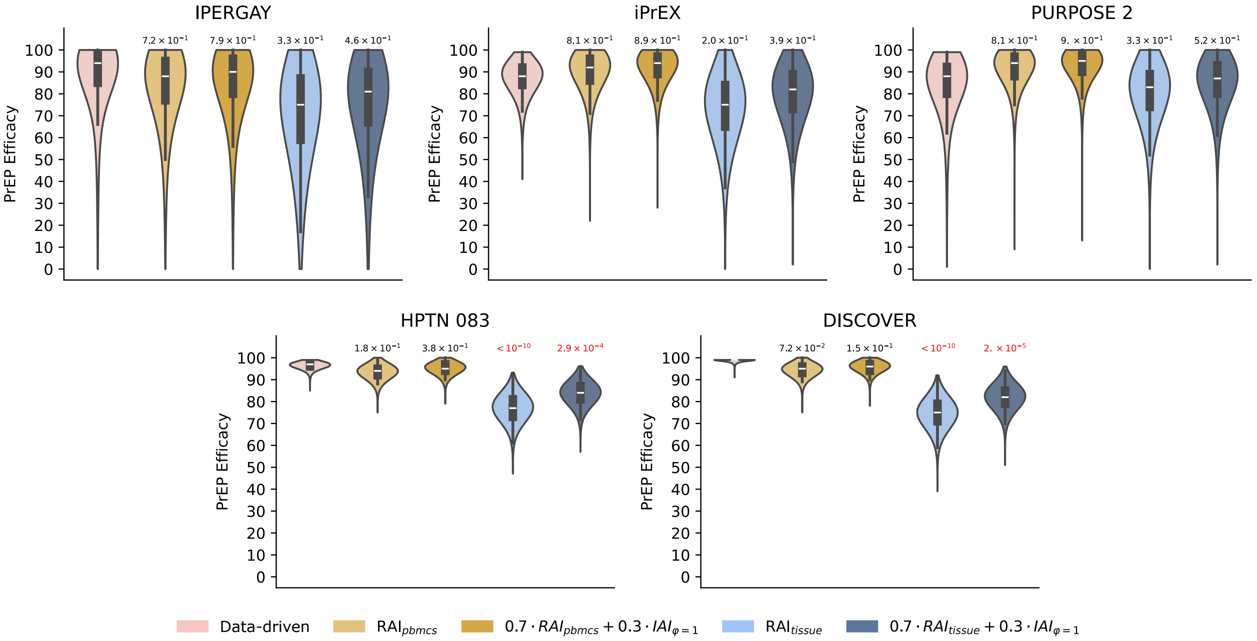


**Figure 5: Sensitivity of findings with regards to virus exposure routes in MSM.** Light orange- and blue violin plots show model-predicted PrEP efficacy distributions for each clinical trial when either PBMC or colorectal tissue concentrations were used to predict PrEP efficacy, assuming only viral exposures via receptive anal intercourse (RAI) exposure (as in the main manuscript). Darker-shaded violins represent a synthetic "extreme-case" scenario designed to maximize the impact of IAI on predicted efficacy: in these simulations, 30% of HIV transmissions are attributed to IAI (see Supplementary Table 5) and are assumed to be fully prevented by PrEP. $P$-values shown above each violin refer to differences in clinical trial outcomes (number of infected individuals) between the data-driven and hypothesis driven approaches under the null hypothesis ($\mathcal{H}_0$: identical distributions) versus the alternative ($\mathcal{H}_1$: different distributions), based on $10^5$ simulations per pair. Source data are provided as a Source Data file.

## Computation of average trial efficacy considering different exposure routes

The average PrEP efficacy in a clinical trial simulation was calculated as the weighted sum of route-specific efficacies $\varphi_{\mathrm{k}}$, according to their relative contribution to HIV infection in the absence of drugs $\mathrm{P}(\mathrm{k} \mid \mathrm{Inf}, \varnothing)$:

$$\hat{\varphi} = \sum_{k} \mathrm{P}(\mathrm{k} \mid \mathrm{Inf}, \varnothing) \cdot \varphi_{\mathrm{k}} \tag{2}$$

where $k \in \{\mathrm{RAI}, \mathrm{IAI}\}$ and $\varphi_{\mathrm{k}}$ are the efficacies sampled from the distributions shown in Figure 1, which can also account for a mixture of both local and systemic pharmacokinetics. This weighting can be derived directly from the definition of efficacy for each exposure type:

$$\varphi_k = 1 - \frac{\hat{P}(\text{Inf} \mid k, \text{Drug})}{\hat{P}(\text{Inf} \mid k, \varnothing)} \tag{3}$$

Explicitly considering the different exposure types, the total efficacy can be expressed as:

$$\hat{\varphi} = 1 - \frac{\pi_{\text{RAI}}\hat{P}(\text{Inf} \mid RAI, \text{Drug}) + \pi_{\text{IAI}}\hat{P}(\text{Inf} \mid IAI, \text{Drug})}{\pi_{\text{RAI}}\hat{P}(\text{Inf} \mid RAI, \varnothing) + \pi_{\text{IAI}}\hat{P}(\text{Inf} \mid IAI, \varnothing)} \tag{4}$$

where $\pi_{\text{RAI}} + \pi_{\text{IAI}} = 1$. Using Eq. (3) to solve for $\hat{P}(\text{Inf} \mid k, \text{Drug})$ gives:

$$\hat{P}(\text{Inf} \mid k, \text{Drug}) = \hat{P}(\text{Inf} \mid k, \varnothing)(1 - \varphi_k) \tag{5}$$

Substituting Eq. (5) into Eq. (4) yields:

$$\hat{\varphi} = 1 - \frac{\pi_{\text{RAI}}\hat{P}(\text{Inf} \mid RAI, \varnothing)(1 - \varphi_{RAI}) + \pi_{\text{IAI}}\hat{P}(\text{Inf} \mid IAI, \varnothing)(1 - \varphi_{IAI})}{\pi_{\text{RAI}}\hat{P}(\text{Inf} \mid RAI, \varnothing) + \pi_{\text{IAI}}\hat{P}(\text{Inf} \mid IAI, \varnothing)} \tag{6}$$

By splitting the numerator, we can identify the posterior probabilities for RAI and IAI:

$$\begin{aligned}\hat{\varphi} = 1 - \Bigg[&\frac{\pi_{\text{RAI}}\hat{P}(\text{Inf} \mid RAI, \varnothing)(1 - \varphi_{RAI})}{\pi_{\text{RAI}}\hat{P}(\text{Inf} \mid RAI, \varnothing) + \pi_{\text{IAI}}\hat{P}(\text{Inf} \mid IAI, \varnothing)} \\ &+ \frac{\pi_{\text{IAI}}\hat{P}(\text{Inf} \mid IAI, \varnothing)(1 - \varphi_{IAI})}{\pi_{\text{RAI}}\hat{P}(\text{Inf} \mid RAI, \varnothing) + \pi_{\text{IAI}}\hat{P}(\text{Inf} \mid IAI, \varnothing)}\Bigg]\end{aligned} \tag{7}$$

where the posterior probabilities are

$$\begin{aligned} P(\text{RAI} \mid \text{Inf}, \varnothing) &= \frac{\pi_{\text{RAI}}\,\hat{P}(\text{Inf} \mid RAI, \varnothing)}{\pi_{\text{RAI}}\,\hat{P}(\text{Inf} \mid RAI, \varnothing) + \pi_{\text{IAI}}\,\hat{P}(\text{Inf} \mid IAI, \varnothing)}, \\ P(\text{IAI} \mid \text{Inf}, \varnothing) &= \frac{\pi_{\text{IAI}}\,\hat{P}(\text{Inf} \mid IAI, \varnothing)}{\pi_{\text{RAI}}\,\hat{P}(\text{Inf} \mid RAI, \varnothing) + \pi_{\text{IAI}}\,\hat{P}(\text{Inf} \mid IAI, \varnothing)}. \end{aligned} \tag{8}$$

Finally, the exposure type-weighted efficacy can be written as a posterior-weighted sum of the route-specific efficacies:

$$\begin{aligned}\hat{\varphi} = 1 - [(1 - \varphi_{RAI})\text{P}(\text{RAI} \mid \text{Inf}, \varnothing) + (1 - \varphi_{IAI})\text{P}(\text{IAI} \mid \text{Inf}, \varnothing)] = \\ = \text{P}(\text{RAI} \mid \text{Inf}, \varnothing) \cdot \varphi_{\text{RAI}} + \text{P}(\text{IAI} \mid \text{Inf}, \varnothing) \cdot \varphi_{\text{IAI}}\end{aligned} \tag{9}$$

## References


[1] Landovitz RJ, Donnell D, Clement ME, Hanscom B, Cottle L, Coelho L, et al. Cabotegravir for HIV Prevention in Cisgender Men and Transgender Women. New England Journal of Medicine. 2021;385(7):595-608.

[2] Marzinke MA, Grinsztejn B, Fogel JM, Piwowar-Manning E, Li M, Weng L, et al. Characterization of human immunodeficiency virus (HIV) infection in cisgender men and transgender women who have sex with men receiving injectable cabotegravir for HIV prevention: HPTN 083. The Journal of Infectious Diseases. 2021;224(9):1581-92.

[3] Castillo-Mancilla JR, Zheng JH, Rower JE, Meditz A, Gardner EM, Predhomme J, et al. Tenofovir, emtricitabine, and tenofovir diphosphate in dried blood spots for determining recent and cumulative drug exposure. AIDS Research and Human Retroviruses. 2013;29(2):384-90.

[4] Ibrahim ME, Castillo-Mancilla JR, Yager J, Brooks KM, Bushman L, Saba L, et al. Individualized adherence benchmarks for HIV pre-exposure prophylaxis. AIDS Research and Human Retroviruses. 2021;37(6):421-8.

[5] Devanathan AS, Dumond JB, Anderson DJ, Moody K, Poliseno AJ, Schauer AP, et al. A novel algorithm to improve PrEP adherence monitoring using dried blood spots. Clinical Pharmacology & Therapeutics. 2023;113(4):896-903.

[6] Johnson MM, Jones CE, Clark DN. The Effect of Treatment-Associated Mutations on HIV Replication and Transmission Cycles. Viruses. 2022;15(1):107.

[7] Gele T, Gouget H, Dimant N, Furlan V, Collins J, Scholz EMB, et al. Whole-body distribution of tenofovir, emtricitabine and dolutegravir in non-human primates. J Antimicrob Chemother. 2024;79(9):2213-20.

[8] Moore M, Glidden D, Anderson P, Hendrix C, Dimitrov D. Dosing forgiveness of oral PrEP for cisgender women remains uncertain. J Int AIDS Soc. 2025;28(5):e26496.

[9] Duwal S, Sunkara V, von Kleist M. Multiscale Systems-Pharmacology Pipeline to Assess the Prophylactic Efficacy of NRTIs Against HIV-1. CPT Pharmacometrics & Systems Pharmacology. 2016;5(7):377-287.

[10] Patel P, Borkowf CB, Brooks JT, Lasry A, Lansky A, Mermin J. Estimating per-act HIV transmission risk: a systematic review. AIDS. 2014;28(10):1509-19.

[11] Sullivan PS, Salazar L, Buchbinder S, Sanchez TH. Estimating the proportion of HIV transmissions from main sex partners among men who have sex with men in five US cities. AIDS. 2009;23(9):1153-62.

[12] Zhang L, Iannuzzi S, Chaturvedula A, Irungu E, Haberer JE, Hendrix CW, et al. Model-based predictions of protective HIV pre-exposure prophylaxis adherence levels in cisgender women. Nature Medicine. 2023;29(11):2753-62.
